# CPU-GPU hybrid platform for efficient spiking neural-network simulation

**DOI:** 10.1186/1471-2202-14-S1-P328

**Published:** 2013-07-08

**Authors:** Francisco Naveros, Niceto R Luque, Jesús A Garrido, Richard R Carrillo, Eduardo Ros

**Affiliations:** 1 Dept. of Computer Architecture and Technology, University of Granada, Granada, Spain; 2 Dept. of Brain and Behavioral Sciences. University of Pavia, Consorzio Interuniversitario per le Scienze Fisiche della Materia (CNISM), Pavia, Italy

## 

Nowadays, research in computational neuroscience is progressively demanding both detailed biologically-plausible neuron models and, at the same time, the simulation of large-scale neural networks in order to better understand the operation of specific nervous circuits of the central nervous system. To that aim, several neural simulators have been developed during last decades; these simulators have been conceived to either simulate detailed neuron models within small-scale neural networks (NEURON [1] and GENESIS [2]), or to simulate neuron models with low degree of biophysical detail within large-scale neural networks (Brian [3] and NEST [4]). In view of this situation, it would be desirable to go a step further in simulating neural networks and combine fast-and-simple neural models with detailed biologically-plausible neurons within large-scale neural networks.

To achieve this goal, we have integrated in our generic neural simulator (EDLUT) [5] two simulation methods (time driven and event driven) with two different hardware processing architectures (CPU and GPU). (For an overview about current types of simulation strategies and algorithms the reader is referred to Brette [6]). It is well known that traditional CPU architectures present good performance when simulating small-scale neural network, however, they still present some drawbacks in case of large-scale simulations. To avoid CPU-based architecture limitations, we have enhanced our simulator efficiency by making it capable of running in a hybrid CPU-GPU platform. In this on-trend CPU-GPU platform, processors can operate conjointly to accomplish a real-time simulation taking full advantage of GPU high performance and CPU versatility. In this work we present how our neural network simulator EDLUT has been upgraded implementing most of those techniques referred in [5] making it capable of being configured in an hybrid event-and-time-driven simulating architecture (results show that using this architecture, up to 50 times higher speed-up values are obtained than using a stand-alone CPU approach).

Once relatively-large-scale simulations can be achieved, the detailed biological neuron models to be simulated become fundamental. These neuron model simulations are intrinsically associated to their techniques of numerical integration. In other words, while numerical integration methods such as Euler and Runge-Kutta are computationally efficient and suitable for neuron models with non-stiff dynamics, more complex numerical integration methods, such as backward differentiation formula (BDF), are usually required for more complex neuron models. We have extended EDLUT with the ability of dealing with complex neural models and also with detailed network characteristics such as spike propagation delays. This point has been revealed as a key factor for learning laws based on spike-timing dependent plasticity.

To sum up, we present here an event-and-time-driven neural network simulator in a hybrid CPU-GPU platform that can simultaneously use simple or complex integration methods.
